# Pain and quality of life evaluation in patients with localized epidermolysis bullosa simplex

**DOI:** 10.1186/s13023-017-0666-5

**Published:** 2017-06-28

**Authors:** Jennifer Brun, Christine Chiaverini, Caroline Devos, Stéphanie Leclerc-Mercier, Juliette Mazereeuw, Emmanuelle Bourrat, Annabel Maruani, Stéphanie Mallet, Claire Abasq, Alice Phan, Pierre Vabres, Ludovic Martin, Christine Bodemer, Sylvie Lagrange, Jean-Philippe Lacour, the Research Group of the French Society of Pediatric Dermatology

**Affiliations:** 1 Reference Centre for Inherited Epidermolysis Bullosa, Archet 2 Hospital, University of Nice Sophia Antipolis, Nice, France; 2 INSERM, U1081, CNRS, UMR7284, Institute for Research on Cancer and Aging, Nice (IRCAN), University of Nice Sophia Antipolis, Medical School, Nice, France; 3 Department of Algology, Archet 2 Hospital, University of Nice Sophia Antipolis, Nice, France; 4 Reference Centre for Cutaneous Rare Diseases (MAGEC), Necker Enfants Malades Hospital, University Paris Descartes, Institut Imagine, APHP, Paris, France; 5 Reference Centre of Rare Diseases of the Skin, Larrey Hospital, Toulouse, France; 6 Reference Centre for Cutaneous Rare Diseases (MAGEC), Saint-Louis Hospital, Paris, France; 7 Department of Dermatology, CHRU de Tours, Tours, France; 8 Department of Dermatology, |La Timone Hospital, Marseille, France; 9 Department of Dermatology, CHRU de Brest, Brest, France; 10 Department of Dermatology, Claude Bernard-Lyon 1 University and Hospices Civils de Lyon, Lyon, France; 11 Department of Dermatology, Dijon University Hospital, Dijon, France; 12 Department of Dermatology, Angers University Hospital, Angers, France

**Keywords:** Localized epidermolysis bullosa simplex, Neuropathic pain, Quality of life

## Abstract

**Background:**

A localized form of epidermolysis bullosa simplex (EBS-l) is considered one of the mildest forms of epidermolysis bullosa (EB), with blisters limited to the palms and soles. However, these lesions can be very painful. The aim of the study was to characterize pain in patients with EBS-l and evaluate its impact on quality of life (QoL). Patients were contacted via the Research Group of the French Society of Pediatric Dermatology and the association of EB patients (DEBRA France). One investigator used a standardized questionnaire that included validated scales for pain and QoL for a telephone interview.

**Results:**

We included 57 patients (27 children). All patients had pain: the mean pain on a 10-mm visual analog scale was >5 for most adults (90%) and children ≥8 years old (94%) when blisters were present and for most adults (73%) and about half of the children ≥ age 8 (53%) during dressing changes. Similar results were found for younger patients. Overall, 75% of patients had neuropathic pain; for 55% of children and 73% of adults, the pain had a moderate to severe impact on QOL. Only seven patients used premedication before changing dressings and seven regularly used oral treatment for chronic pain. A total of 21% and 23% of patients used non-steroidal anti-inflammatory drugs and grade 2 analgesics, respectively. These treatments were not effective for neuropathic pain. Six patients tried 5% lidocaine plasters on their feet, with good efficacy.

**Conclusions:**

EBS-l patients have frequent and severe pain with neuropathic characteristics. This pain is undertreated and affects QoL.

## Background

Inherited epidermolysis bullosa (EB) is a heterogeneous group of rare genodermatoses characterized by cutaneous and/or mucosal fragility resulting in post-traumatic blistering. The diseases are classified according to the level of skin cleavage. In EB simplex (EBS), the cleavage plane is within the basal keratinocytes of the epidermis [1]. EBS is the most frequent form of EB, with a worldwide prevalence of 1/35,000 to 1/150,000 [2]. Many patients carry dominant mutations in K5 or K14 genes encoding for keratins 5 and 14, respectively, mostly expressed in the epidermal basal layer. The localized form of EBS (EBS-l) is considered one of the mildest forms of EB, with blisters localized to palms and soles. However, although this form is benign, skin lesions can be painful.

Pain is constant in EB [3]. Although it has been well assessed in patients with the most severe forms of EB, little is known about pain in patients with milder forms such as EBS-l. We conducted a multicenter observational study in France to assess the presence, characteristics and impact on quality of life of pain in EBS-l.

## Methods

Patients >6 months old of both sexes with EBS-l were included between May 2015 and January 2016. They were analyzed by age 6 months to 16 years (children) and >16 years (adults). EBS was diagnosed clinically by a dermatologist of one of the French Reference or Competence Centers for cutaneous rare diseases. Immunofluorescence analysis of skin biopsy and/or molecular analysis were not necessary for inclusion. Patients were contacted by the Research Group of the French Society of Pediatric Dermatology, the EB patient association (DEBRA France) and social networks of DEBRA France.

After telephoning patients about the study, information and consent notes were sent by e-mail. After obtaining written consent, a standardized questionnaire was sent by e-mail to patients and/or their parents before telephone interviews, to familiarize them with it, then patients were interviewed by telephone by one investigator (JB). The questionnaire included scales to evaluate pain and quality of life (QoL). An estimated level of EB-related pain was assessed in adults and in children aged 8 and over using a linear visual analog scale (VAS) [4], ranging from 0 (no pain) to 10 (unbearable pain). To do so, we asked to patients to “indicate the intensity of their pain on a scale of 0 (pain) to 10 (worst pain imaginable)”. Parents of children between 4 and 7 years completed the behavioral Face, Legs, Arms, Cry, Consolability (FLACC) scale [5] and the self-assessment Faces Pain Scale (FPS) [6]. FLACC is an observational tool for quantify pain behaviors. Facial expression, leg movement, activity, cry, and consolability are each scored 0–2, for a total FLACC score of 0–10. The FPS is a self-report measure of pain intensity developed for children. Pain intensity ranges from 0 (no pain) to 10 (unbearable pain) depending on faces expression. Children under 4 years were evaluated only by the FLACC scale completed by their parents. These scores were used to assess the intensity of daily pain, and pain during flares of blisters and dressing changes.

The Pain Questionnaire of Saint Antoine (QDSA) [7] was used to identify the characteristics of pain and its intensity. This questionnaire has 60 word descriptors categorized into 17 subgroups including nine sensory groups, seven affective groups and one evaluative group. The patients pick the word descriptors and score them from zero (not at all) to four (extremely).

The neuropathic pain diagnostic questionnaire (DN4 questionnaire) [8] can confirm, with a high level of reliability, the notion of a neuropathic component to the chronic pain affecting the patient. It’s a 10-item questionnaire divided into seven questions for the patient and three items related to the clinical conducted by the physician. A 7-item interview shortened version, with a 3-item threshold value has been reported. Each item of this questionnaire assesses the presence of subjective symptoms or objective signs of neuropathic pain.

The Hospital Anxiety and Depression (HAD) scale [9] was used to assess anxiety and depressive disorders. The questionnaire features seven questions for anxiety and seven for depression with a respective maximal score of 21. A score ≤ 7 means absence of symptomatology, from 8 to 10: doubtful symptomatology and ≥11: certain symptomatology.

QoL was assessed by the Children’s Dermatology Life Quality Index (cDLQI) [10] for children (4–16 years) and the Quality of Life in EB questionnaire (QOLEB) [11] for adults. The cDLQI contains 10 questions relating to experiences during the previous week. The 10 questions cover six areas of daily activities including symptoms and feelings, leisure, school or holidays, personal relationships, sleep and treatment. Each item is scored from 0 (not at all) to 3 (very much) for a total score ranging from 0 (absence of impact on QoL) to 30 (major impact on QoL).

The QOLEB is a specific scale developed for EB patients. It consists of 17 individual items which were scored from 0 to 3 in the order of least to most impact. The questions broadly fell into two dimensional constructs: ‘functioning’ (walk, eat, write,…) and ‘emotions’. The total score range from 0 (absence of impact on QoL) to 51 (major impact on QoL).

This study has been considered as a non-interventional observational study, and then no ethics committee approval was necessary.

## Results

**Fig. 1:**
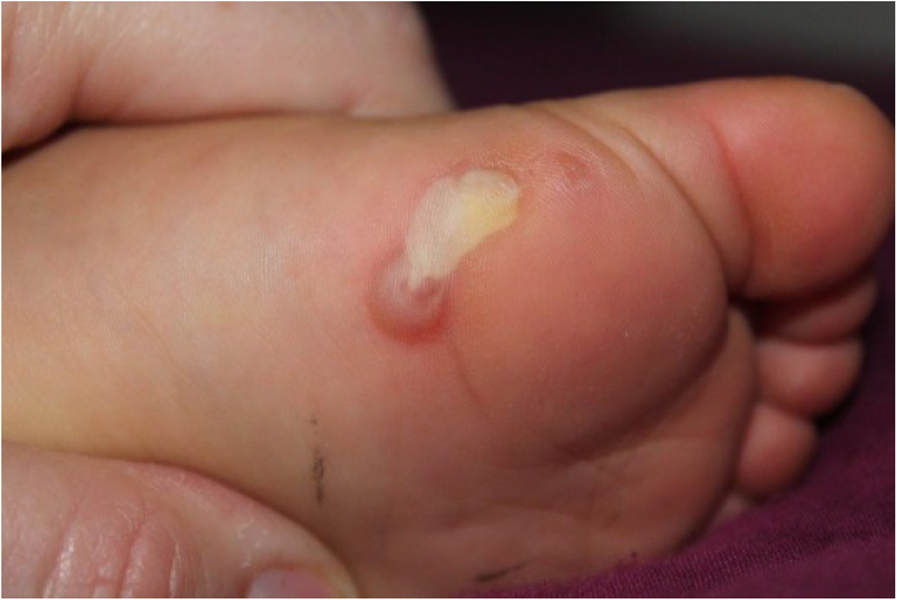
Blisters on the sole in a child

We included 57 patients (total sex ratio [M:F] 1:3). The mean age for the 27 children was 8.5 ± 4.6 years (range 21 months to 16 years) and for the 30 adults 38.4 ± 16.2 years (range 17–85 years). Nine patients had de novo EBS-l. As expected, patients declared to have blisters localized to palm and soles without involvement of nails, mouth, eyes and nutritional compromise. The mean age at the beginning of blisters was 11.4 months (range birth to 20 years). Blistering was preferentially localized to palms and soles **(**Fig. 1 **)**.

### Patients had moderate to severe pain during blister flares and dressing changes

All patients declared having pain. Pain was localized to feet (100%) and hands (39%) and was usually associated with the formation of blisters and care. Five patients had pain even without blisters. Pain occurred before (10%), during (51%) or after (39%) the onset of blisters. As expected, it was triggered by friction (100%), walking (95%), heat (42%), trauma (39%) and hyperhidrosis (33%). Summer was the worst season, with 79% of patients experiencing constant or frequent pain and 56% unable to walk more than 1 km because of pain, as compared with 40% and 21%, respectively, during the winter.

For daily pain, 24% of children ≥8 years old and 20% of adults reported a mean cutaneous pain >5 on the VAS, and 50% of children ≤7 years old a pain ≥4 by the FLACC and FPS.

**Fig. 2:**
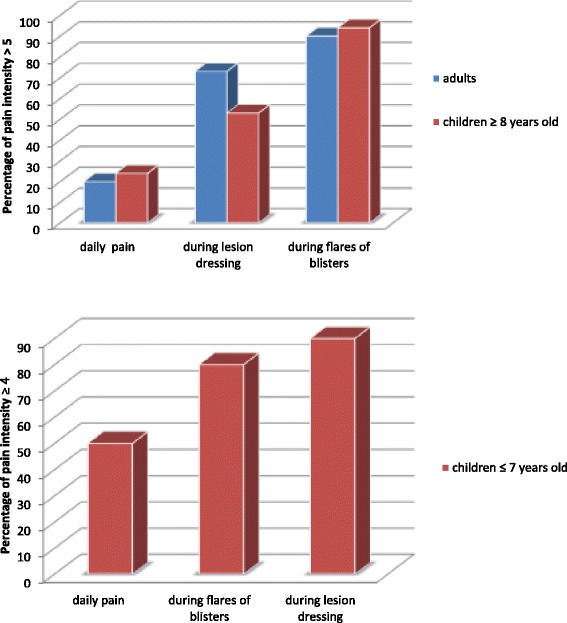
Assessment of the level of epidermolysis bullosa (EB)-related pain - daily pain, during flares of blisters and lesion dressing- for adults (*n* = 30) and children (*n* = 27)^*^. ^*^Pain was assessed by a linear visual analog scale (VAS) for both adults and children ≥8 years old. Children 4 to 7 years old were evaluated by the behavioral Face, Legs, arms, Cry, Consolability (FLACC) scale and the Faces Pain Scale (FPS)

The formation of blisters and erosions secondary to blisters was painful, even at a distance from care. For 90% of adults and 94% of children ≥8 years old, the mean cutaneous pain was >5 on the VAS. The mean pain intensity was 7 ± 1.7 and 6.9 ± 1.9, respectively. For 80% of children ≤7 years old, the mean cutaneous pain was ≥4 and the mean pain intensity 6.8 ± 2.7 by the FLACC and FPS (Fig. 2).

During dressing changes, for 73% of adults and 53% of children ≥8 years old, the mean cutaneous pain was >5 on the VAS and mean pain intensity 6 ± 2.5 and 5.4 ± 2, respectively on the VAS*.* For 90% of children ≤7 years old, the mean cutaneous pain was ≥4 and mean pain intensity 6.4 ± 2 by the FLACC and FPS (Fig. 2).

### Patients had neuropathic pain

Overall, 75% of patients (19 children and 24 adults) had neuropathic pain according to the DN4 questionnaire, with a total score ≥ 3. This pain was described as “burning” (88%), “pricking” (67%), “electric shocks” (33%),“tingling” (44%), “numbness” (37%) and “itching” (86%) (Table 1). These results were confirmed by the QDSA. Furthermore, 93% of patients complained of itching exclusively in feet during blister healing, and 59% of children and 43% of adults declared waking at night because of the pain.

**Table 1:** Frequency of sensory descriptors according to DN4 questionnaire (neuropathic pain diagnostic questionnaire)

	*n* (%) patients	intensity/4 (mean)
Positive score: DN4	43 (75)	
Variables		
« burning »	38 (88)	3,1
« painful cold »	3 (7)	1,5
« electrics shocks »	14 (33)	2,6
« tingling »	19 (44)	2,9
« pricking (picks and needles) »	29 (67)	2,7
« numbness »	16 (37)	1,5
« itching »	37 (86)	2,4

### EBS-l affected QoL

For 55% of children and 73% of adults, pain had a moderate to severe impact on their QoL, according to the cDLQI and QOLEB, respectively (Table 2). For adults, the mean QOLEB score was 6.6 ± 4.9/51. More specifically, 87% of patients felt frustrated, 27% embarrassed, 17% depressed, 33% uncomfortable, and 40% anxious or worried by their disease. EB affected patients in their relationship with their friends (4%) and family (47%). In all, 87% of patients could not participate in sport, 60% were markedly or severely affected in their ability to move outside their house, and 80% were affected in their ability to go shopping. Furthermore, 43% were financially affected by their EB. According to the localized form of EB, no patient had eating or bathing difficulties**.**

**Table 2:** Quality of life evaluation (QoL) (cDLQI: Children’s Dermatology Life Quality Index, QOLEB: quality of life in EB questionnaire)

Variables	Number	Percent
Children (cDLQI)		
0–1 = no fact	1	5
2–6 = mild	11	55
7–12 = moderate	6	30
13–18 = severe	3	15
19–30 = major impact	2	10
Adult (QOLEB)		
0–4 = very mild	3	10
5–9 = mild	5	17
10–19 = moderate	18	60
20–34 = severe	4	13
35–51 = major impact	0	0

For children with EBS-l, the mean cDLQI score was 8.1 ± 5.1/30. For 76%, QoL was affected by pain and 56% felt sad. In total, 52% of children had to decrease or stop any physical activity because of pain and 68% had to wear adapted shoes; 36% declared spending a bad holiday or having school difficulties because of pain; 32% felt a change in relationships with their friends and 24% were the target of teasing by classmates. In total, 52% had difficulty sleeping because of pain.

### EBS affected socioprofessional issues

In France, some chronic and severe diseases can be 100% covered by the health insurance system upon special request. However, only 56% of our patients (21 children and 11 adults) were covered. As well, only 10% (4 adults and 2 children) were considered disabled.

In all, 22 adults had a job, 4 were disabled workers, 5 were students and 3 were housewives; 35% had occasional work stoppage because of their pain and 2 patients had to change their work because of pain. Overall, 87% of children were absent from school because of pain: once a week (6%), once or twice a month (13%), 5 to 10 absences a year (39%) and fewer than 4 absences a year (29%). A patient had to quit school because of too frequent absenteeism and psychological impact. A protocol for care at school had (http://context.reverso.net/traduction/anglais-francais/care+protocol+has) been established for only 11 children (41%).

Overall, 10% of patients (1 child and 5 adults) had a regular follow-up by a psychologist because of the disease. According to the HAD, 11% of patients (2 children and 4 adults) were considered to have anxiety because of the disease and 4% of adults had depression.

### Treatment of pain

Only 7 patients had a specific follow-up by an algologist, all in a French Reference Center. Only 7 used analgesics before changing the dressing: 5 used the eutectic mixture of local anaesthetics (EMLA®) applied on blisters 1 h before a dressing change and 2 children used tramadol (Table 3). For pain independent of care, 63% of patients used paracetamol, often by self-medication, 50% reporting poor efficacy; 21% of patients used non-steroidal anti-inflammatory drugs (NSAIDs, mainly ibuprofen), with good efficacy. Only 23% of patients (5 children and 8 adults) used a grade 2 analgesic (moderate opioids, tramadol or codeine), with good efficacy, but half experienced side effects. One adult used a tricyclic antidepressant for neuropathic pain and 6 patients with itching and neuropathic pain tried 5% lidocaine plasters on their feet, with good efficacy. In all, 33% of patients (13 adults and 6 children) did not use any treatment. Four patients had botulinum toxin injections: 2 (1 adult and 1 child) had injections twice a year, with good results, 1 patient did not tolerate the pain during the procedure and 1 patient, despite a decrease in blister number, experienced an increase in pain lasting 3 months after the injections.

**Table 3:** Therapeutic management of pain (NSAIDs: non-steroidal anti-inflammatory drugs)

Variables	Number			Side effects	efficacy yes, n	Treatment compliance, n
	Total	Children	Adults			
Oral treatment						
Grade 1 analgesic						
Paracetamol	36	20	16	1	18	2
NSAIDs	12	2	10	2	12	0
Grade 2 analgesic						
Moderate Opioids	3	0	3	2	2	0
Tramadol	5	4	1	1	5	3
Codein Paracetamol	5	1	4	2	4	1
Analgesic for neuropathic pain						
Tricyclic antidepressant (Amitriptyline)	1	1	0	0	1	1
Anxiolytic						
Hydroxyzine (Atarax^®^)	1	1	0	1	0	0
Local treatment						
lidocaine 2.5% and prilocaine 2.5% (EMLA^®^)	5	4	1	0	2	2
5% lidocaine plasters (VERSATIS^®^)	6	3	3	0	6	4
No treatment						
Oral	19	6	13			
Local	46	19	27			

Only 26% of patients (9 adults and 6 children) wore specific shoes particular, in particular shoes with a silicone sole. Only eight patients (3 children and 5 adults) were followed by a podologist; 13% were followed by paramedical professionals (physiotherapist or osteopath) for back pain secondary to bad position when walking. In all, 9% of patients (1 child and 4 adults) were practicing breathing techniques or relaxation techniques and 2 patients attended medical spas. Four patients used alternative medicine. 30% of children used distraction (tablet or video games) to ameliorate the pain during dressing changes.

## Discussion

EBS-l is usually considered one of the mildest subtypes of EB. However, most of our patients with EBS-l had frequent and severe pain during blister flares and dressing changes. We found only one article on this topic, by Fine et al. [12], who reported a median daily pain score > 5 on a VAS for 22.7% of children and 17.8% of adults with EBS-l. Our results were similar. However, when the pain was assessed during blister onset or during dressing changes, these data were higher. This point is of importance. Patients with EBS-l do not have blisters every day during the year and usually do not have other reasons for pain, which explains that the median daily pain is not the best tool to assess their pain.

Analysis of pain characteristics by the DN4 questionnaire showed that 75% of patients had neuropathic pain. Furthermore, 93% of patients had pruritus during the healing of blisters, a frequent symptom associated with neuropathic pain, and 49% of patients experienced night waking. Neuropathic pain is frequent in EB patients, independent of the subtype, but few data on this topic are available. [13–16] Indeed, ours is the first study to evaluate neuropathic pain with specific questionnaires in EBS patients. Because neuropathic pain results from irritation of a peripheral nerve [17] and the epidermis is rich in nerve endings, particularly in soles, our results are not surprising [8].

No specific data are available on QoL in patients with EBS-l. Fine et al. [12] analyzed QoL for patients with all subtypes of EBS, including severe and generalized forms: only 2% of patients were totally dependent during activities of daily life (bathing, grooming, and walking) and 14.4% of adults and 8.3% of children had walking or standing limitations due to cutaneous pain [12]. In our study, 58% of patients were unable to walk more than 1 km because of pain during summer and only 21% in winter, which confirms that a global assessment of these patients is not relevant. Consistent with the localized involvement, no patients had limitations in other activities such as eating or bathing.

The questionnaire QOLEB [11] is the first QoL tool developed specifically to evaluate the QoL in adults with EB. Since its publication, a few studies [11, 18, 19] have analyzed QoL in EBS adult patients, with mean scores ranging from 7.9 ± 5.3/51 [19] to 13.7 ± 8.7/51. For children, we found only one study that evaluated QoL by the cDLQI, with a mean score of 15/30 [9]. However, none of these studies had specific data on EBS-l patients. The inclusion of a generalized subtype of EBS could explain the lower mean QoLEB and cDLQI scores in our study than in other studies [10, 11, 18, 19]. For 73% of our adults with EBS-l, pain had a moderate to severe impact on QoL (QOLEB), as compared with 81% of adults with inherited ichthyosis (IQoL-32 scale), another severe genodermatosis [20]. Children with EBS-l experience impaired QoL equivalent to that caused by psoriasis and atopic dermatitis according to the cDLQI [21].

Consistent with the impact of EBS-l on QoL, patients had also socioprofessional difficulties. Overall, 35% of our adults had occasional work stoppage due to their pain, two patients had to change their work because of pain and 87% of children were absent from school because of pain. However, only 4/30 adults were considered disabled workers and 11% of children had a specific protocol for pain and care at school. Furthermore, only 48% of patients were totally covered by the health insurance system. These results can be explained by the misinformation of patients themselves and their physicians, an underestimation of their symptoms and a misunderstanding of the disease by social professionals. Thus, follow-up by social assistance staff, possibly in a specialized center, seems essential.

Recently, the best clinical practice guidelines for pain in EB [22] were published. Therefore, the management of disease for our patients should be improved. Only 26% of our patients used preventive measures to reduce the onset of new lesions by wearing flexible, seamless shoes with internal seams and using protective padding. No patient used silver-lined or silk socks for anti-friction action and reducing the risk of superinfection [23]. In the same way, despite the frequency and the intensity of pain during treatment, only 10% of patients used premedication with topical anaesthetics (EMLA® cream, lidocaine 2.5% and prilocain 2.5%) and/or tramadol, which have good efficiency, before changing dressings. Other topical treatments such as morphine mixed in hydrogel formulations and amethocaine were not used. Interestingly, 63% of patients used oral treatment for chronic pain of their feet, but only 18% used it regularly. Paracetamol used in first intention was less effective than NSAIDs and tramadol. Only 3 patients were taking opioids for severe pain and 12% of patients had already consulted a pain specialist. Reasons of such results are complex and need further investigations. However, some explanations can be hypothesized: a fatalism of many patients in front of this familial and incurable disease, their fear of pain treatment, the absence of correct information of patients and general practitioners and the absence of available evaluation of pain and QoL in this specific EB subpopulation leading to probable under estimation of pain’s patients by EB specialists used to treat more severe forms.

Psychological therapies (distraction, visualization, imagery/virtual reality and breathing techniques) for pain management have been shown to modify pain intensity, reduce related distress, decrease pain-related functional disability and improve coping with pain [24]. In our study, 30% of children used distraction (tablet or video games) to ameliorate the pain and 9% of patients were practicing breathing techniques or relaxation techniques. This is an interesting perspective for pain management in EB.

Finally, only a few patients used specific treatment for neuropathic pain. Six patients used 5% lidocaine plasters in this indication, with good efficiency and no side effects. Lidocaine exerts its action by blocking the abnormal sodium channels present in higher number on hyperactive or damaged nociceptors that transmit pain. To our knowledge, this is the first report of the efficiency of a local analgesic in this indication. Further controlled studies are necessary to demonstrate the interest of 5% lidocaine plasters in this indication, because topical treatment for a localized disease is attractive.

Botulinum toxin injections were reported to be efficient in EBS-l patients in a case series [25]: 13 patients (93%) reported reduced plantar blistering and pain, and the improvement score was ≥4 for 4/6 patients with EBS. The mean effect duration was 3 months. The rationale for using botulinum toxin in keratinopathies is to inhibit a sweat-induced maceration of the fragile epidermis, thus reducing plantar blistering and pain. In our study, four patients had injections; 2 (1 adult and 1 child) had twice-a-year injections, with good results [26]. The main difficulty was pain during the treatment procedure. In our study, one child received propofol and 1 adult topical lidocaine under occlusion, oral opioids and nitrous oxide. Once again, this treatment is interesting but needs to be evaluated in a controlled study.

## Conclusion

We found that EBS-l patients have frequent and severe pain during blister flares and dressing changes and that this pain has neuropathic characteristics. This pain is undertreated but has an impact on QoL and socioprofessional activities. A better knowledge of the specificities of pain in EBS-l is essential for diagnosis and adapted treatment, with the development of new strategies including topical analgesic treatments such as lidocaine patch*.* A close collaboration between the dermatologist and algologist is essential to evaluate and manage pain.

## Abbreviations

cDLQI: Children’s dermatology life quality index
DN4 questionnaire: Neuropathic pain diagnostic questionnaire
EB: Inherited epidermolysis bullosa
EBS: EB simplex
EBS-l: Localized form of epidermolysis bullosa simplex
FLACC: Behavioral face, legs, arms, cry, consolability
FPS: Faces pain scale
HAD: Hospital anxiety and depression
QDSA: Pain questionnaire of saint antoine
QoL: Quality of life
QOLEB: Quality of Life in EB questionnaire
VAS: Linear visual analog scale
